# Out of Equilibrium? The World’s Changing Ice Cover

**DOI:** 10.1289/ehp.119-a20

**Published:** 2011-01

**Authors:** Charles W. Schmidt

**Affiliations:** **Charles W. Schmidt**, MS, an award-winning science writer from Portland, ME, has written for *Discover Magazine, Science,* and *Nature Medicine.*

In August 2010 an iceberg four times the size of Manhattan broke off Greenland’s northwestern coast and began drifting out to the sea. At nearly 100 square miles, this was the largest iceberg to appear in Arctic waters since 1962 and a fresh indicator that Greenland’s frozen landscape is undergoing significant changes.^1^

Originally part of the much larger Petermann glacier, which flows down from Greenland’s interior into a coastal fjord, the iceberg detached for unknown reasons. It could be that ordinary glacial dynamics were at work, and indeed, Petermann glacier “calves” (breaks off) icebergs routinely, although generally not ones so large as this. But the unusual size of the iceberg also might signal a response to global warming, and that’s what worries scientists.

Accelerated melting of the glaciers and other frozen masses that make up Earth’s cryosphere has become a widespread phenomenon. Apart from what’s happening in Greenland, coastal glaciers also are thinning off West Antarctica, where an enormous ice sheet containing enough water to raise sea level by more than 20 feet has attracted growing concern. Meanwhile, a vast percentage of the world’s land-based glaciers also are in retreat, according to Richard Armstrong, a senior research scientist at the National Snow and Ice Data Center (NSIDC) at the University of Colorado, Boulder.

“There’s a lot of regional variation, so there are some exceptions to the trend,” Armstrong says. “But in general, the Earth is warming, and glaciers are shrinking in most areas.” Likewise, the human health implications of the world’s changing ice cover vary regionally.

## No Simple Answers

Glacial retreat is often described as global warming’s most visually compelling manifestation. But the influence of climate on the cryosphere doesn’t lend itself to simple generalizations. Glaciers, ice sheets, and seasonal snowpack are part of an exceedingly complex climate system with many unknowns.

Beata Csatho, an associate professor of geophysics at the State University of New York at Buffalo, says glaciology is just now emerging from an observational phase dominated by data collection. Scientists have only now begun to model these data to project future trends, she says. But uncertainty about how the cryosphere responds to climate change can be frustrating to those in the public who want definitive answers to either support or reject assumptions about global warming. That puts glaciologists in an uncomfortable position, pushed to supply conclusions prematurely and besieged by misconceptions.

Skeptics who cite one or two advancing glaciers as evidence that humans don’t have an impact on climate are likely overstating their case, says Jeffrey Kargel, a University of Arizona glaciologist. The near-unanimous scientific consensus—including the opinion even of most climate change skeptics—is that anthropogenic greenhouse gas emissions have likely accelerated an ongoing warming trend, although by how much isn’t clear.^2^ The global mean temperature increased 0.74°C from 1906 to 2005,^3^ and average Northern Hemisphere temperatures during the past 60 years likely are the highest of the past 1,300 years, if not longer.^4^

Some scientists are concerned that if warming continues, the global meltdown could cause catastrophic effects. Sea level is already rising at an accelerated rate—a global average of 0.1 inch per year between 1993 and 2003, according to the United Nations Intergovernmental Panel on Climate Change (IPCC), nearly twice the average annual rise over the longer period of 1961 to 2003.^3^ More drastic increases could displace millions of people.

The IPCC has projected a rise in sea level of anywhere from 7 inches to 2 feet by the end of this century.^3^ A 2-foot rise would eliminate 10,000 square miles of U.S. coastline, destroy low-lying marshes and wetlands, contaminate freshwater aquifers with intruding salt water, and increase vulnerability to storm surges.^5^ A 3-foot rise over the next century, recently projected by another group,^6^ would flood coastal cities around the world.

## Arctic Changes

The fastest warming rates are in the Arctic, where temperatures are increasing at twice the global average.^7^ Arctic waters off Greenland’s western coast have warmed since the 1990s, at the same time that scientists have documented extensive “thinning,” or a steady loss of mass from the island’s glaciers and also its interior ice sheet, which is thought to contain about 8–10% of the world’s fresh water by various estimates.^8^ Should that retreating pattern continue, scientists say, sizeable portions of Greenland’s ice sheet might eventually drain enough water into the ocean to raise sea level by up to 23 feet, enough to submerge London and Los Angeles.^9^

According to Walt Meier, a research scientist at the NSIDC, high Arctic warming rates can be explained by a reflective phenomenon known as albedo: melting sea ice exposes dark ocean surfaces, which absorb sunlight instead of reflecting it back into space. Oceans retain that warmth and then transfer it back to the atmosphere. “And that sets up a vicious cycle,” Meier explains. “More melting, followed by more ocean warming, followed by more warming of the air.”

The consequences are clearly evident in satellite imagery that shows Arctic sea ice decreasing by about 9% per decade since 1979. In September of that year, Arctic sea ice covered slightly more than 3 million square miles, an area roughly equal to that of the lower 48 states, Meier says. By September 2010, the sea ice had dwindled to roughly 1.8 million square miles.^10^

Meanwhile, climate change appears to exert a mysterious influence on how Arctic winds and ocean currents interact, says Hamish Pritchard, a scientist with the British Antarctic Survey in Cambridge, UK. And as Arctic winds change in direction and speed, he says, they’re somehow drawing warmer ocean currents closer to Greenland’s coast, where glaciers that buttress the island’s massive ice sheet spill into the sea.

Glaciers that gain as much mass from snow as they lose from calving icebergs are said to be in equilibrium. But if calving dominates over the accumulation of new mass, which Pritchard says is the case in Greenland now, then seawater can intrude where coastal glaciers attach themselves to land. “Ice always melts when it’s in contact with seawater, and that erodes the glacier’s frontal regions,” Pritchard says. Eric Rignot, an earth scientist at the University of California, Irvine, recently documented that Greenland’s coastal glaciers are melting 100 times faster at their floating end points than they are on land.^11^

At the same time, warming air is degrading the island’s ice sheet from above. Around 10 years ago, scientists discovered that “moulins,” or narrow channels in the ice sheet, were draining unexpectedly large amounts of summer meltwater from the surface of the ice toward the underlying ground. And as that water accumulates below the ice sheet, sections of the ice lift off the ground and start flowing rapidly toward the coast, explains Gordon Hamilton, an associate research professor at the University of Maine, Orono.

The IPCC did not consider this “moulin effect” when, in its 2007 assessment report, it predicted that sea level might rise by as much as 2 feet by 2099.^3^ Models that do consider faster flows of ice to the coast will raise those projections, but by how much isn’t yet clear, Hamilton says.

## Antarctic Changes

Summer meltwater isn’t currently so much an issue in Antarctica, which is so far south that the majority of its ice sheet stays frozen all year. (Much of Greenland, on the other hand, resides at latitudes shared by Norway.) Separated from West Antarctica by the Trans-Antarctic Mountains, the East Antarctic ice sheet is the largest ice mass on the planet, contains enough water to raise sea level by almost 200 feet. But East Antarctica’s ice sheet rests on an enormous landmass above present-day sea level and is generally considered stable, according to Claire Parkinson, a climatologist with the National Aeronautics and Space Administration.

The West Antarctic ice sheet, however, rests on a landmass grounded roughly a mile below sea level, meaning that if the ice sheet wasn’t there, it would be replaced by a deep ocean. And that makes it vulnerable to encroaching seawater that could speed its disintegration.^12^ Coastal ice shelves (portions of the ice sheet that hang out over the oceans) buttress the seaward flow of ice from the continent, but geological history suggests a drastic change is possible. “According to limited evidence, the West Antarctic ice sheet likely disintegrated during one or more of the interglacial periods within the Pleistocene epoch, the latter lasting from nearly two million to about twelve thousand years ago,” Parkinson says.

Both of West Antarctica’s biggest ice shelves—namely, the Ross ice shelf to the west and the Ronne-Filchner ice shelf to the east—appear stable at this point, Parkinson says. Yet according to Pritchard, glaciers along West Antarctica’s midpoint are thinning at an accelerated pace: the Pine Island glacier is thinning by up to 18 feet per year, the neighboring Smith glacier by 27 feet per year, and the Thwaites glacier by 12 feet per year.^13^

Other nearby glaciers also are thinning from what Ted Scambos, a glaciologist with the NSIDC, says is an influx of deep, warm ocean water pushed toward the coast by changing wind patterns. That dynamic, he says, results in part from the Southern Hemisphere’s ozone hole, which allows for regional warming in the upper atmosphere that, in turn, influences local air currents.^14^

According to Csatho, ice shelf losses in the West Antarctic are partly offset by strong gains from snowfall in some inland areas, so the degree to which the ice sheet might be imperiled isn’t clear. “But if the ice shelves do collapse, then portions of the ice sheet are at greatly increased danger of flowing into the ocean,” Parkinson adds.

Scientists first considered this possibility in 1968, when the late John Mercer, a professor at The Ohio State University, predicted that global warming caused in part by industrial pollution might destroy West Antarctica’s ice shelves and allow its ice sheet to flow toward the coast.^15^ Mercer’s warning seemed prescient when the Larsen ice shelf—which buttresses land ice on the Antarctic Peninsula to the north—began crumbling in 1995. A section called Larsen A disintegrated first, followed seven years later by Larsen B, a nearly 1,300-square-mile mass that disintegrated spectacularly in just over a month after having been there for more than 10,000 years.^10^

The collapsing Larsen ice shelves had no effect on sea level, given that they were already floating and displacing water, according to Scambos. What’s significant is that when the ice shelves disappeared, land-based glaciers behind them began moving faster toward the coast, just as Mercer predicted. Scambos says there isn’t enough land ice on the Antarctic Peninsula to raise sea level appreciably. The Thwaites, Pine Island, and Smith glaciers are a different story, however, given that the West Antarctic ice sheet—with its capacity for a more catastrophic rise in sea level—lies behind them.

## What Does It Mean for People?

No one knows how high the global sea level will rise in coming decades or how many people will be affected, although small island nations and coastal areas are expected to bear the brunt of the impact.^16^,^17^ But there is evidence that glacial retreat is already affecting human populations through an increase in so-called glacier lake outburst floods (GLOFs).^18^

Excessive glacial runoff can pool in downstream areas and create lakes that appear and then disappear from one year to the next. And when these lakes breach, the resulting GLOFs endanger local lives and infrastructure. “In March 2006, an avalanche fell from the Quelccaya ice cap into one of these lakes and created a mini-tsunami that breached a dam and flooded the entire valley below,” says Lonnie Thompson, a glaciologist and professor at The Ohio State University. The Quelccaya ice cap of Peru is the largest tropical ice mass in the world.

Should the Andean glaciers disappear altogether, Thompson adds, Peru’s population could face an opposite problem: significant water shortages. Indeed, the “zero-degree isotherm,” or the altitude at which snow begins to melt, has climbed to higher elevations at earlier time points in spring, reflecting a steadily warming climate.^19^ This means snow and glacial melting occurs sooner than it ordinarily would—a concern for populations that rely on meltwater for seasonal water supplies.

That’s already happening now in Peru, where many populations rely on meltwater from Andean glaciers for domestic and industrial water needs.^18^ In 1978 the Qori Kalis glacier—which extends out from the Quelccaya ice cap—was retreating 18 feet per year.^20^ But in the last 15 years, this rate grew to 18 feet per year, and 25% of the glacier has disappeared since 1978.^20^ “We’re expecting this retreat to accelerate even further,” says Thompson.

Similarly, communities in the Southwestern United States rely on meltwater from the Sierra Nevada snowpack, which forms every year in the mountains that define California’s eastern border. The Sierra Nevada snowpack begins to accumulate in October, peaks around April, and then melts entirely. When rainfall decreases in summer, meltwater makes up the shortfall, draining into reservoirs and contributing a significant fraction to water supplies throughout the Western states.^21^

According to research by Sarah Kapnick, a PhD candidate in atmospheric and oceanic sciences at the University of California, Los Angeles, the Sierra Nevada snowpack has been trending toward an earlier peak by about 0.6 day per decade since 1930, such that it starts melting almost a week sooner now than it did 80 years ago.^22^ Assuming the trend continues or accelerates, she says the peak could arrive yet another one to three weeks sooner by the end of the century. If the peak advances significantly, then meltwater will exceed reservoir capacity and could generate springtime floods while depriving communities of water later in the year, Kapnick says.

Apart from the southwestern United States and Peru, it’s not clear where retreating ice masses could affect local water supplies. It is said that 1.3 billion people in Southern Asia might face a risk, given widespread glacial retreat in the Eastern Himalayas.^23^ Several of the world’s major rivers, including the Ganges, the Indus, and the Yangtze, originate in the Himalayas, so many people who live in the region take it on faith that glacial retreat will reduce downstream waterflow, according to Armstrong. Indeed, the IPCC, in its 2007 report, warned that the Ganges, Indus, and other rivers on India’s northern plain could become seasonal if the Himalayan glaciers were to undergo a catastrophic retreat.^3^

However, in March 2010, Alford and Armstrong completed a study for the World Bank that suggests the IPCC likely overstated its conclusion.^24^ According to their analysis, glacial runoff from the mountains of Nepal, at the headwaters of the Ganges, contributes only about 4% of the total water entering the river every year.^24^ The rest, Armstrong says, comes from seasonal snowmelt and monsoon rains that dominate river flow from June through September. “So even if the Himalayan glaciers disappeared completely, the downstream Ganges flow wouldn’t be significantly affected,” Armstrong says.

Regional variation makes it hard to predict how water supplies originating in the Himalayas will respond to climate change. Even as glaciers retreat in its eastern ranges, those in the west—given that they reside at higher altitudes where temperatures don’t fall below freezing—are stable or advancing. “The wild card is precipitation,” says Armstrong. “Most of the climate models are pretty consistent on temperature increases with rising greenhouse gas [levels], but they fall apart when it comes to precipitation—some show more and others less with climate change depending on location. And if you get more precipitation, it would fall as snow above the freezing level, and you could wind up with increasing glacier mass at higher elevations.”

## Open-Ended Change

Armstrong’s point illustrates an essential problem: although climate change has global consequences, it manifests in different ways depending on regional features. And that complexity leaves interpretations of its cause and future impacts open to debate, even to attack.

But look at the global system as a whole, Scambos emphasizes, and a fundamental truth emerges. “Virtually every glacial region on Earth is in retreat, as are [ice masses] on both of the poles,” he says. And that gives us a consistent picture that hangs together well—this is happening at the same time that the oceans, land, and air are also warming.”

## Figures and Tables

**Figure:**
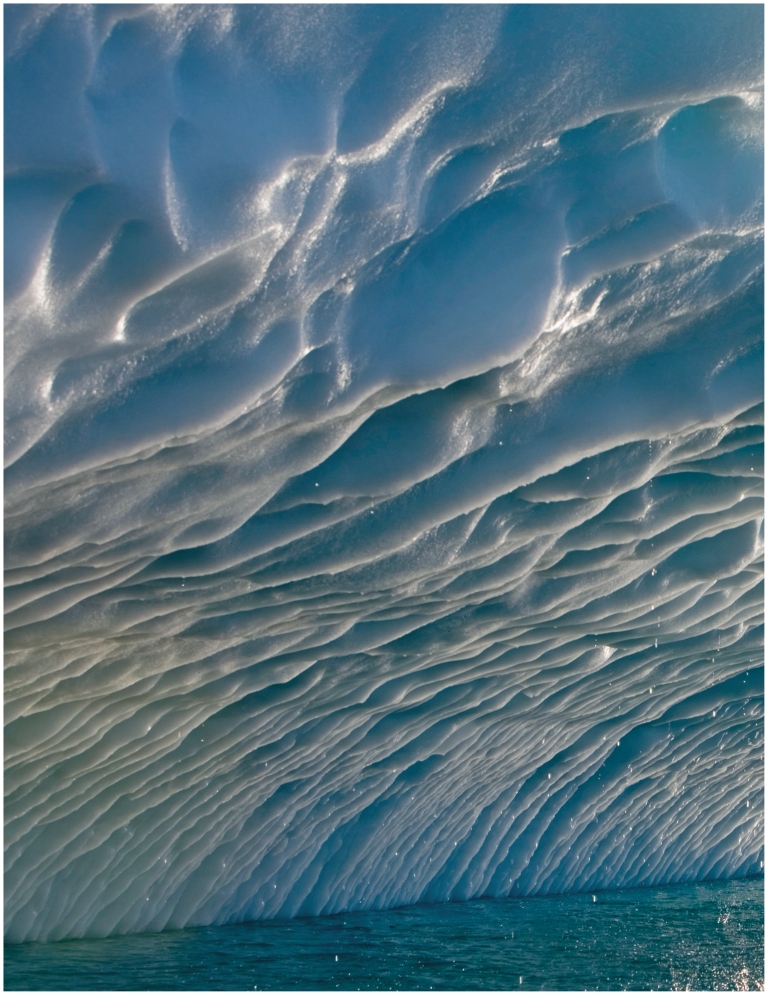
Melting water streams from an iceberg in Ilulis-sat Icefjord, Greenland. The iceberg calved from Sermeq Kujalleq (in Danish: Jakobshavn Isbrae), one of the world’s fastest-moving and most active glaciers. Sermeq Kujalleq nearly doubled its calving rate between 1993 and 2003.^25^

**Figure:**
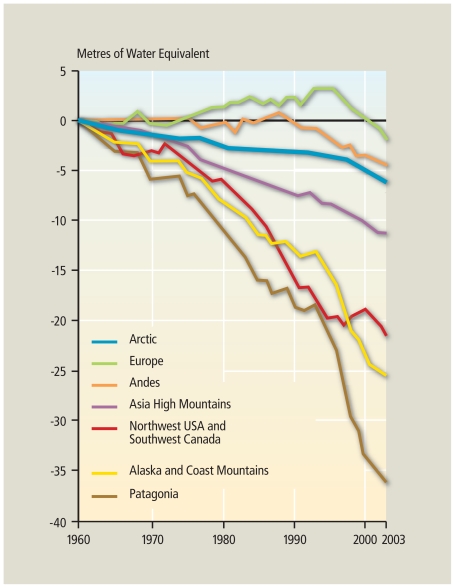
Glacier Changes in Recent Years^18^ Cumulative glacier mass balances for seven mountainous regions show that most mountain glaciers are losing mass, with the overall rate of mass loss accelerating in the past decade.

**Figure:**
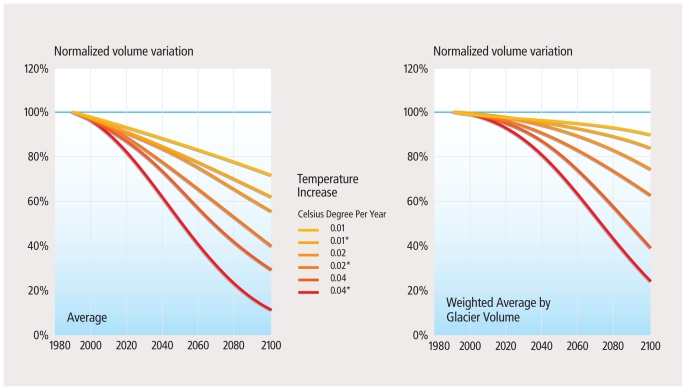
Modeled Glacier Changes^18^ Scaled glacier volume changes reflect a range of climate change scenarios. Labels are in °C per year^−1^, and * indicates a 10% increase in precipitation per degree warming. The lefthand panel shows the average of all model results, and the righthand panel shows results weighted by glacier volume.

**Figure:**
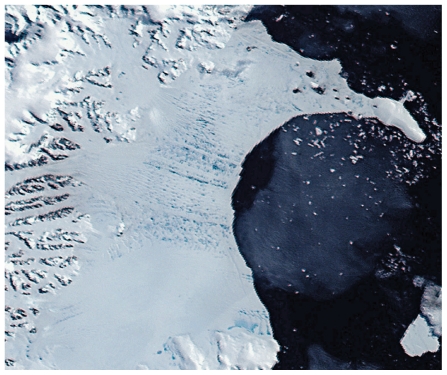
31 Jan 2002 NASA’s MODIS satellite sensor captures true-color images of the Larsen B ice shelf as it begins what would become a spectacular collapse. In this image, dark bluish melt ponds dot the surface of the ice shelf.

**Figure:**
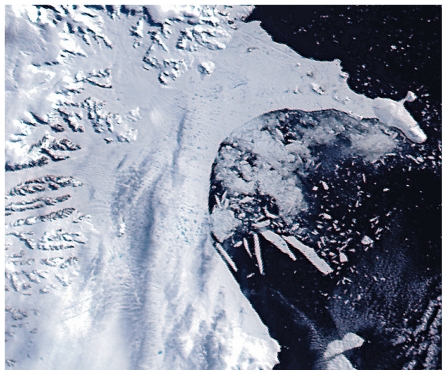
17 Feb 2002 Minor retreat continues; several melt ponds drain through new cracks within the shelf.

**Figure:**
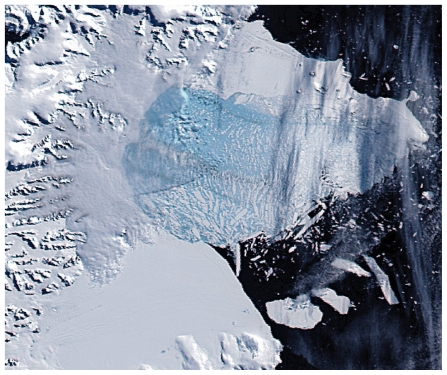
5 Mar 2002 Thousands of sliver icebergs and a large light blue area of very finely floating ice remain where the shelf formerly lay. Brownish streaks within the floating chunks indicate debris exposed from the former underside and interior of the shelf.

**Figure:**
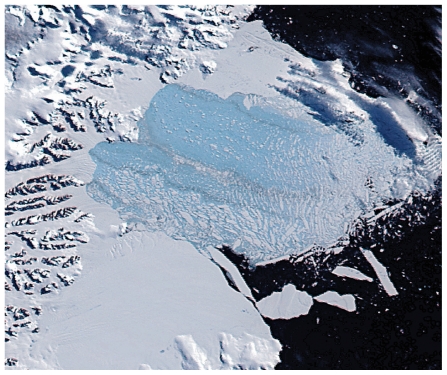
7 Mar 2002 Within just over five weeks a total area of approximately 1,250 square miles collapsed.

**Figure:**
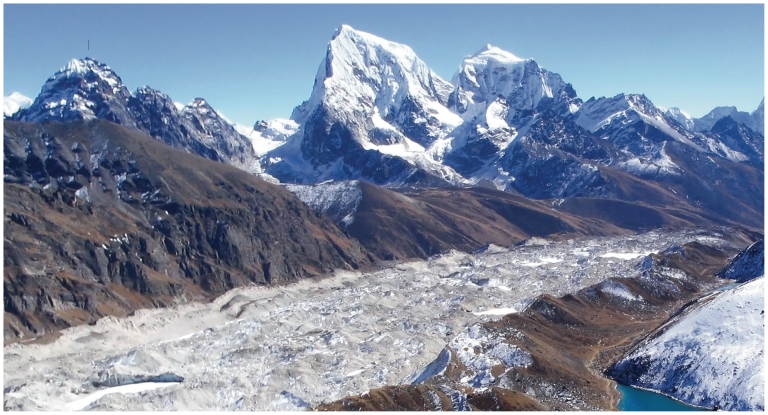
Ngozumpa glacier (above) is the largest glacier in Nepal. Himalayan glaciers have spurred several glacier lake outburst floods, in which lake waters contained by glaciers are suddenly released. Hopar glacier (opposite, top) is located in the Karakoram mountain range of Pakistan. Many glaciers in the Karakoram appear to be either stable or advancing, demonstrating the pivotal role regional variation plays in the changing cryosphere.

**Figure:**
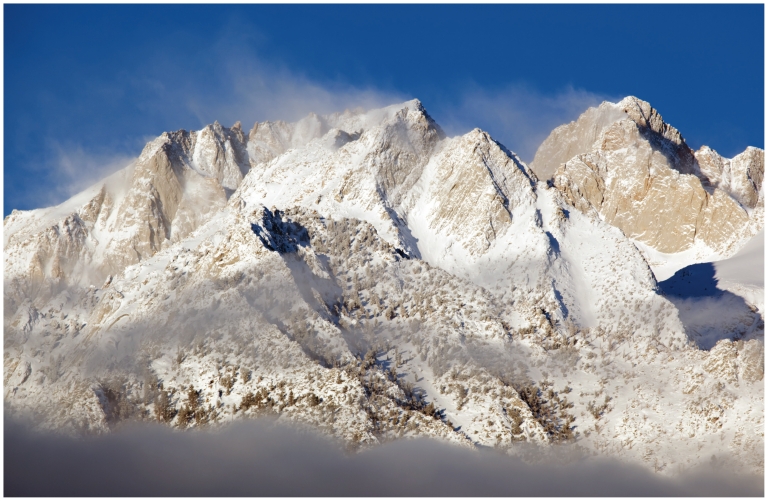


**Figure:**
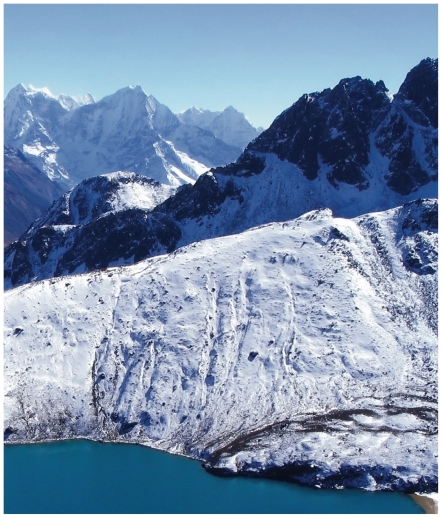


**Figure:**
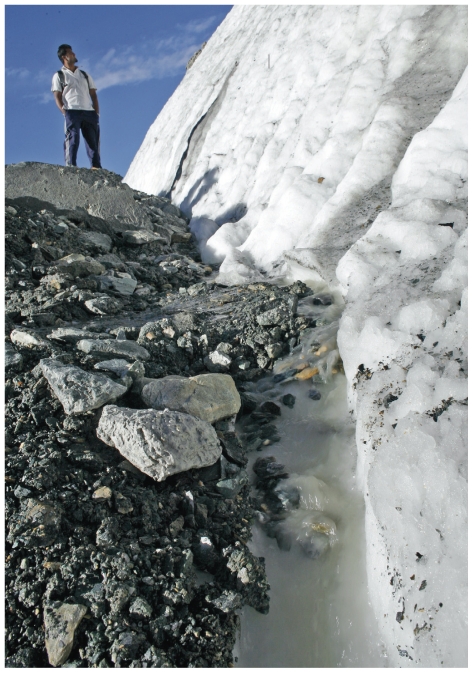


**Figure:**
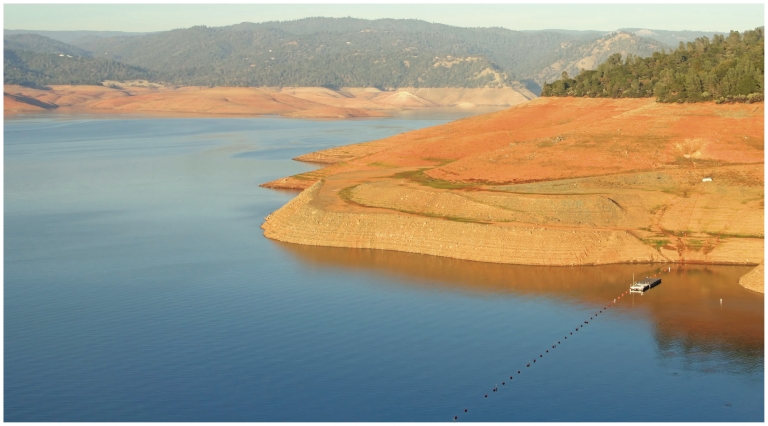
Reservoirs such as California’s Oroville Lake (below, seen during a drought that has persisted the past three years) are fed by snowmelt from the Sierra Nevada (opposite, bottom). The Sierra Nevada snowpack now melts almost a week sooner than it did in 1930. If this trend continues or accelerates, it raises the possibility of increased springtime flooding and drought later in the season.
